# Supplementary material for: A Pipeline for Screening Small Molecule-Enhanced Protein Stability in A Bacterial Orphan Receptor
Source: bioRxiv. 2025 Sep 12:2025.07.03.663076. Originally published 2025 Jul 5. Preprint. [Version 2] doi: 10.1101/2025.07.03.663076 (PMC12236518; doi:10.1101/2025.07.03.663076)
Supplement: Supplement 1 [file media-1.pdf]

Supporting Information

A Pipeline for Screening Small Molecule Enhanced Protein Stability in A Bacterial Orphan Receptor

James J. Siclari, Denize C. Favaro, Richard H. Huang, Kevin H. Gardner

Contents:

Figures S1-S7

Tables S1-S3

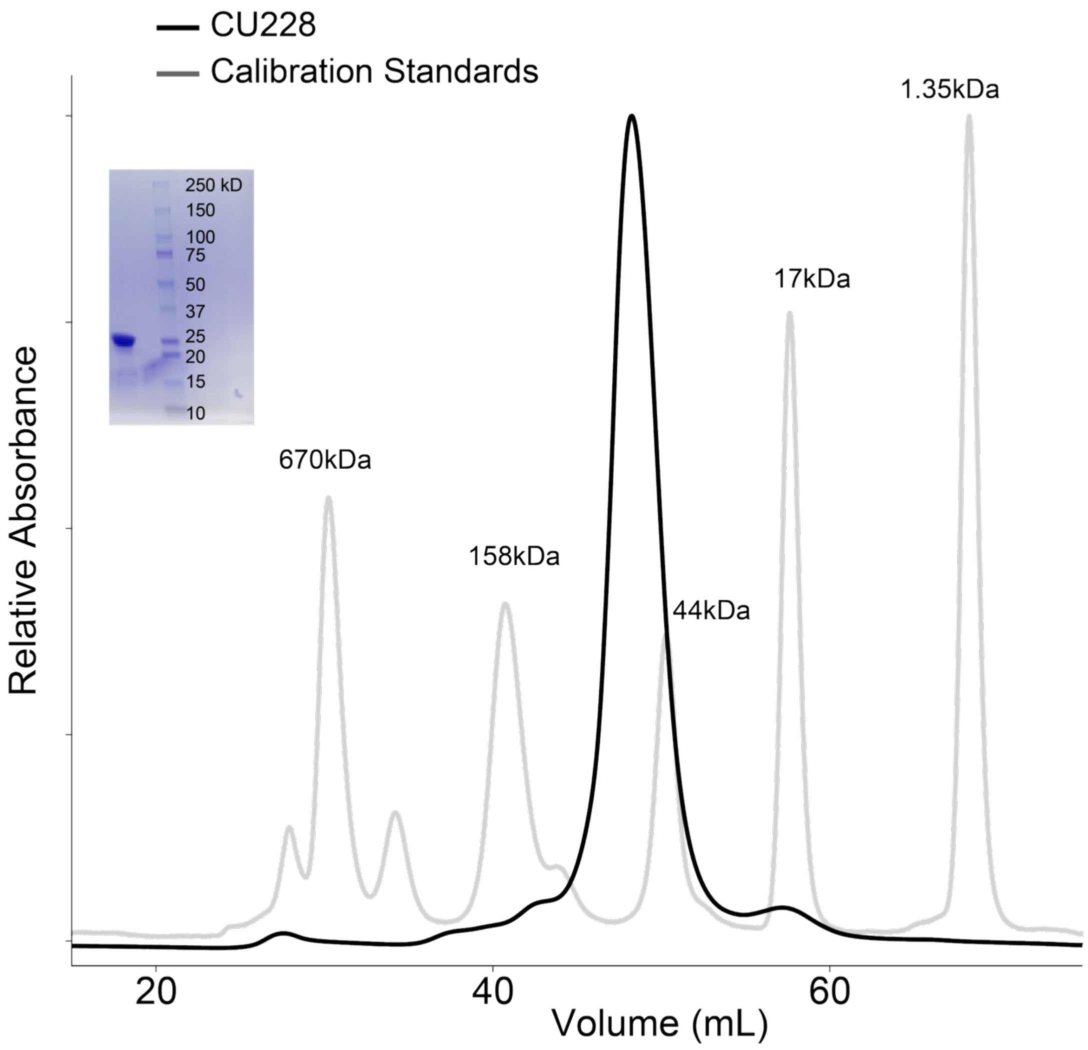

Figure S1. CU228 purification. SEC chromatogram of CU228 (black) and chromatography standards (grey). The insert shows SDS-PAGE gel of purified CU228.

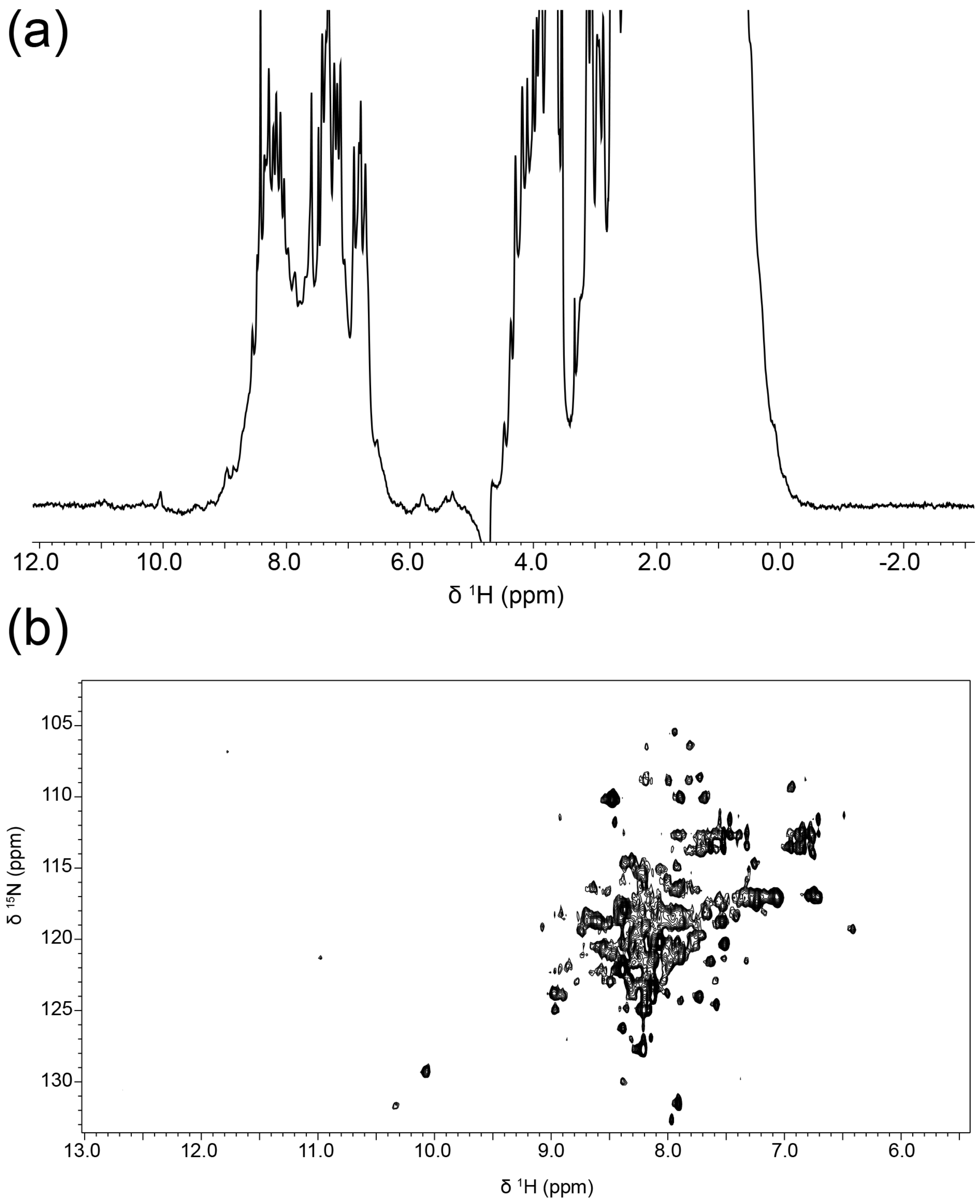

Figure S2. Initial characterization of apo CU228 by 1D and 2D protein NMR. (a) ^{1}H NMR of CU228. (b) ^{15}N/^{1}H TROSY of CU228.

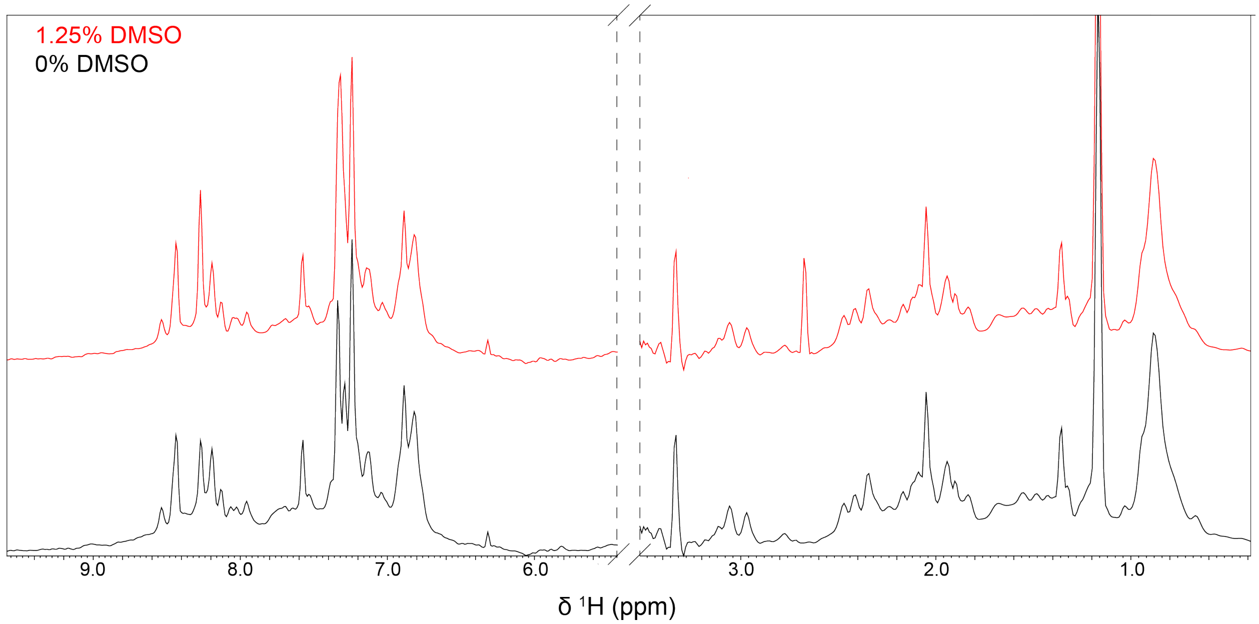

Figure S3. ^{1}H NMR with CU228 in the presence and absence of 1.25% DMSO-d6. Aliphatic and amide sections of a 1D ^{1}H spectra of 136 μM CU228 in the absence (black) or presence (red) of 1.25% DMSO-d6.

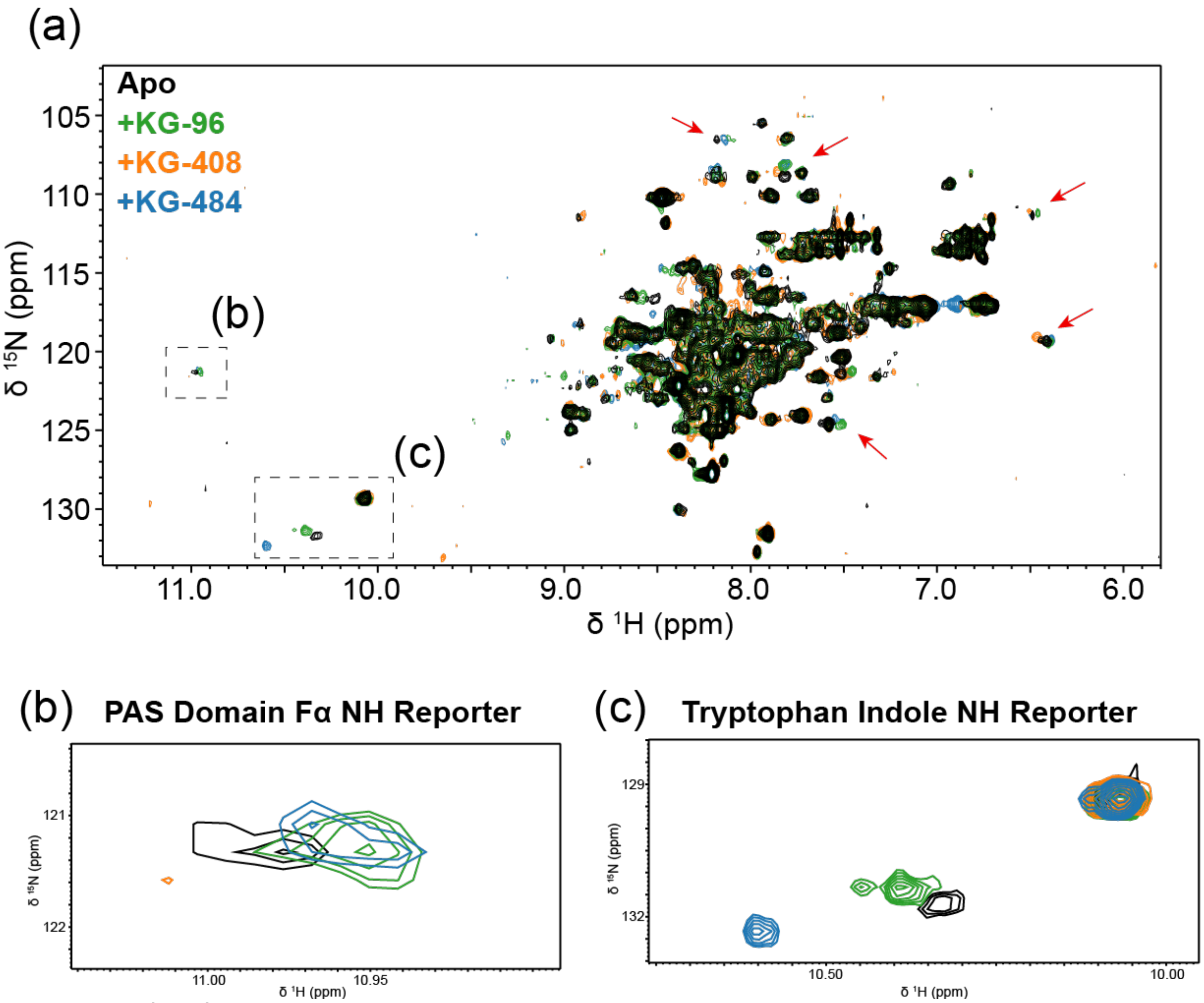

Figure S4. $^{15}N/^{1}H$ TROSY spectra of CU228 alone and the presence of small molecule ligands. (a) TROSY spectra of 165 μM CU228 (black) and with the addition of 1 mM KG96 (green), KG408 (orange), or KG484 (blue). Red arrows indicate peaks showing ligand-dependent chemical shift changes, with specific compounds giving rise to specific patterns of changes. (b) Expansion of spectral region associated with a hallmark PAS domain peak arising from a backbone amide at the N-terminus of the Fα helix as detailed in the text. (c) Expansion of spectral region typically occupied by tryptophan sidechain indole NH groups. Four Trp residues are present in CU228, all of which are located in the PAS domain.

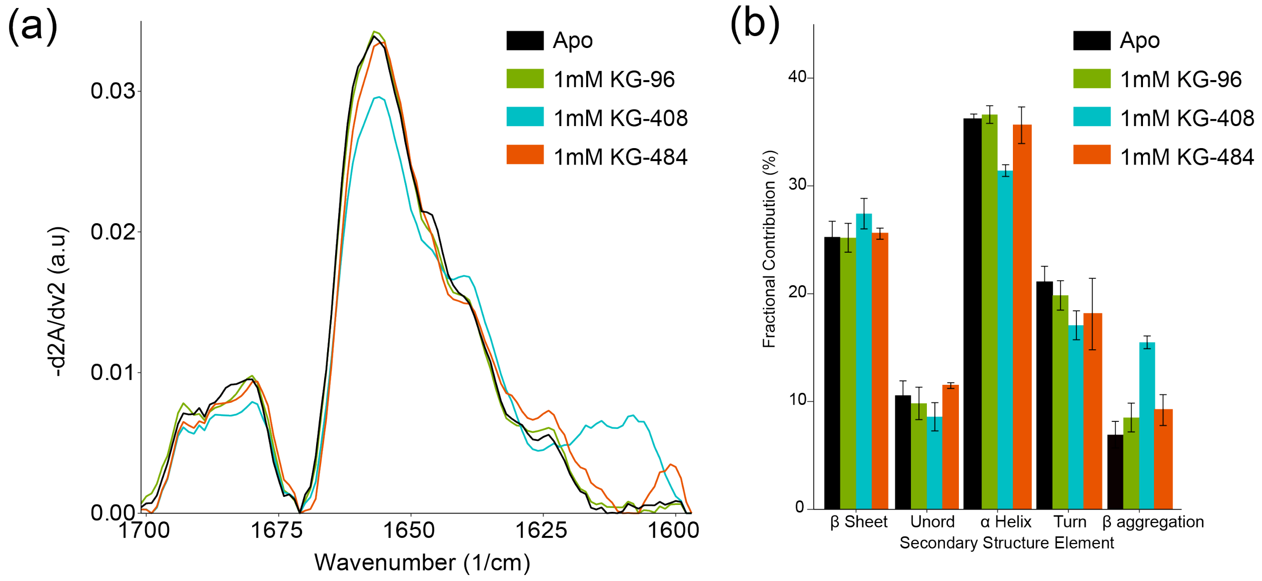

Figure S5. MMS of CU228 with small molecules at 25°C. (a) Second derivative Gaussian fits of IR spectra recorded for Apo CU228 and CU228 with 250 μM KG-96, KG-408, and KG-484. (b) Higher-order structure fractional contribution of each sample calculated from traces in panel a.

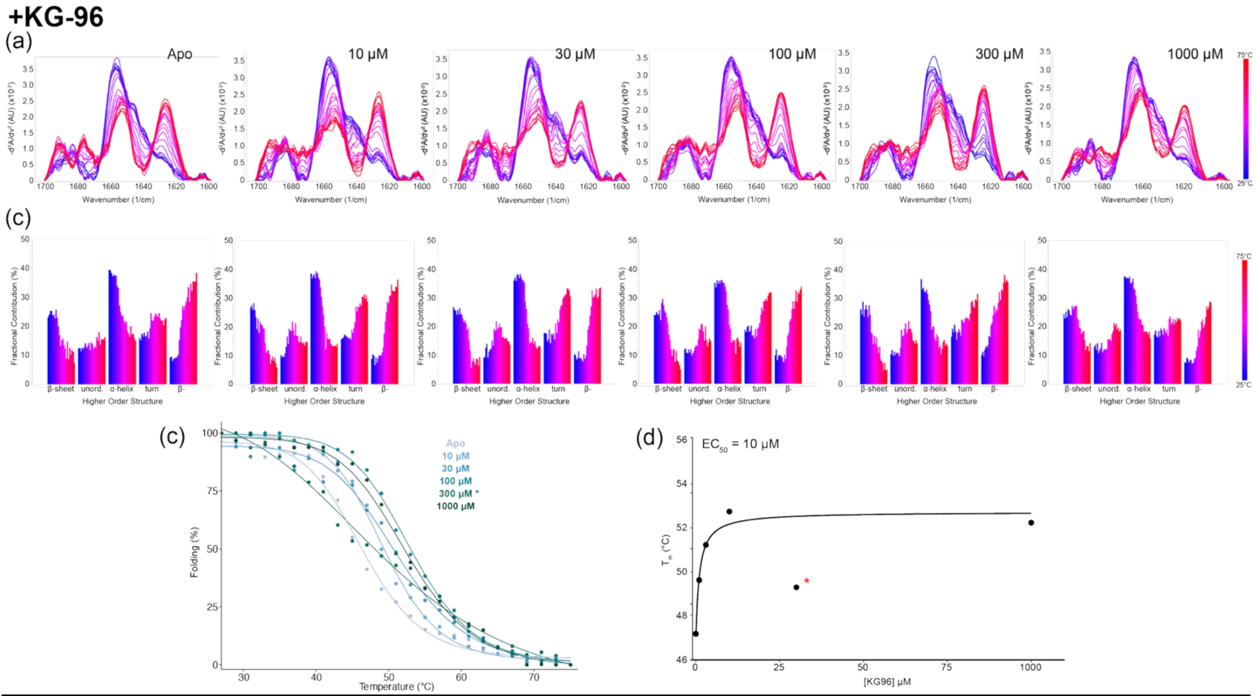

Figure S6. Concentration Dependent Thermal ramp MMS of CU228 and KG-96. (a) IR traces across from 25-75°C at five different concentrations. (b) Higher order structure fractional contribution as a function of temperature for each titration point. (c) Global melting curves of CU228 with addition of each KG-96 concentration. (d) KG-96 impact on the melting temperature of CU288 as a function of ligand concentration. Red asterisk indicates that the 300 µM point was omitted from fitting.

+KG-484

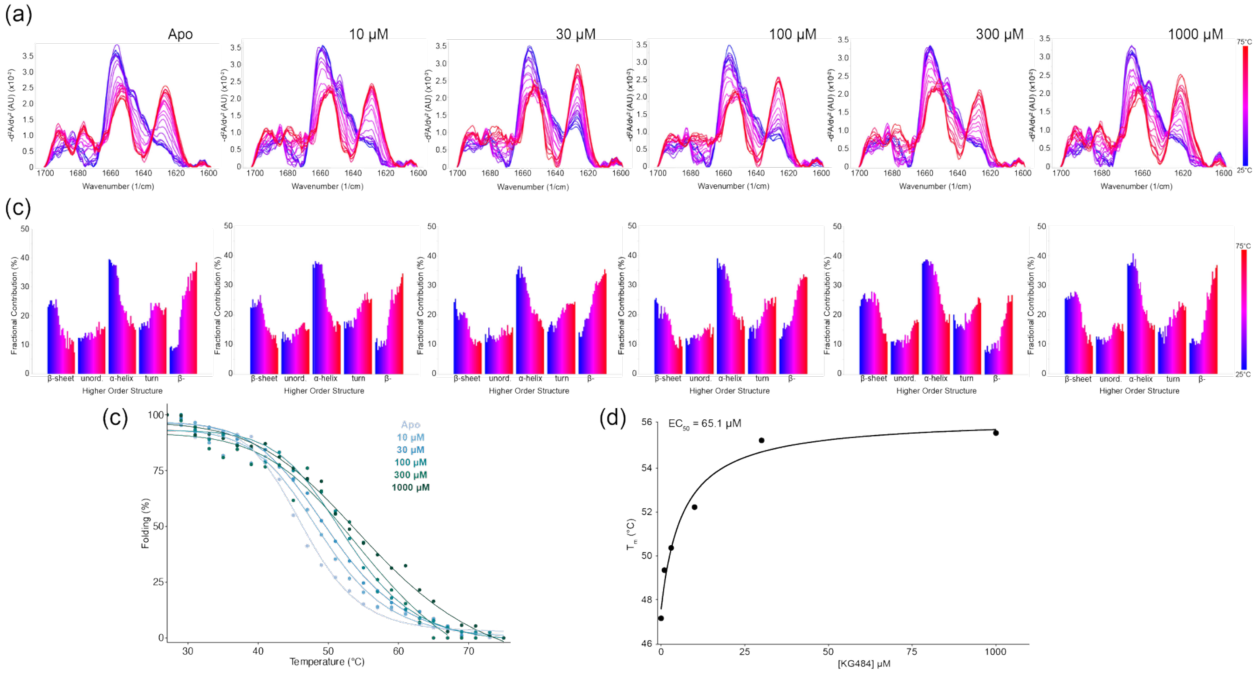

Figure S7. Concentration Dependent Thermal ramp MMS of CU228 and KG-484. (a) IR Traces across from 25-75°C at five different concentrations. (b) Higher order structure fractional contribution as a function of temperature for each titration point. (c) Global melting curves of CU228 with addition of each KG-484 concentration. (d) KG-484 impact on the melting temperature of CU288 as a function of ligand concentration.

Wavenumber (cm^{-1})	Secondary Structure
1618	Beta- (intermolecular β-sheet)
1624	Beta-
1627	Beta-
1632	Beta
1638	Beta
1642	Beta
1650	Unordered
1656	Alpha
1666	Turn
1672	Turn
1680	Turn
1688	Turn
1695	Beta-

Table S1. Peak assignments for Gaussian curve fitting.

Ligand	[Ligand] (μM)	T_m (β-)	T_m (β-sheet)	T_m (α-helix)
**Apo**	-	48.3	47.0	43.3
**KG-96**	10	49.0	49.6	47.2
	30	51.3	52.3	47.2
	100	51.8	54.6	47.3
	300	42.3	53.9	44.1
	1000	53.4	60.0	47.8
**KG-408**	10	48.6	49.2	44.3
	30	51.9	51.8	45.7
	100	51.7	53.3	45.1
	300	53.7	55.4	45.0
	1000	54.4	60.6	46.5
**KG-484**	10	50.5	51.2	46.7
	30	49.4	52.0	47.8
	100	53.4	55.7	51.1
	300	55.7	60.0	52.5
	1000	53.3	61.0	47.5

Table S2: Secondary structure melting temperatures (T_m, in °C) of CU228 with and without small molecule ligands. KG-96, KG-408, and KG-484 were added at five different concentrations as indicated. (β- = intermolecular β-sheet aggregation)

Ligand	Peak Region	[Ligand] (μM)	STD-AF_{max}	k_{sat} (s^{-1})	STD-AF_0 (STD-AF_{max} x k_{sat})
KG96	1	0	-	-	0
		200	1.16	0.0632	0.073312
		400	0.744	0.123	0.091512
		600	1.39	0.066	0.09174
		800	1.22	0.0763	0.093086
		1000	0.52	0.232	0.12064
	2	0	-	-	0
		200	1.75	0.0578	0.10115
		400	1.26	0.11	0.1386
		600	1.9	0.0882	0.16758
		800	1.26	0.142	0.17892
		1000	1.28	0.156	0.19968
	3	0	-	-	0
		200	0.912	0.212	0.193344
		400	1.08	0.187	0.20196
		600	1.65	0.129	0.21285
		800	1.16	0.207	0.24012
		1000	1.18	0.21	0.2478
KG408	1	0	-	-	0
		200	1.21	0.513	0.62073
		400	2.33	0.426	0.99258
		600	3.52	0.336	1.18272
		800	4.09	0.296	1.21064
		1000	4.73	0.327	1.54671
	2	0	-	-	0
		200	1.57	0.505	0.79285
		400	3.03	0.41	1.2423
		600	3.83	0.367	1.40561
		800	4.41	0.362	1.59642
		1000	5.44	0.302	1.64288
	3	0	-	-	0
		200	1.36	0.508	0.69088
		400	2.61	0.43	1.1223
		600	3.57	0.389	1.38873
		800	4.39	0.328	1.43992
		1000	5.27	0.313	1.64951
KG484	1	0	-	-	0
		200	0.0747	0.821	0.0613287
		400	0.326	0.113	0.036838
		600	0.123	0.844	0.103812
		800	0.259	0.308	0.079772

		1000	0.185	0.62	0.1147
	2	0	-	-	0
		200	9.48	0.00214	0.0202872
		400	0.246	0.16	0.03936
		600	1.21	0.0274	0.033154
		800	1.76	0.0254	0.044704
		1000	0.533	0.111	0.059163
	3	0	-	-	0
		200	0.0552	0.276	0.0152352
		400	11.9	0.00178	0.021182
		600	0.178	0.155	0.02759
		800	6.91	0.00418	0.0288838
		1000	0.276	0.152	0.041952

Table S3: Variables derived from STD buildup curves (**Eq. 2**) used to plot Langmuir fittings.
