# Supplementary material for: A Pipeline for Screening Small Molecule-Enhanced Protein Stability in A Bacterial Orphan Receptor
Source: bioRxiv. 2025 Jul 5:2025.07.03.663076. Preprint. [Version 1] doi: 10.1101/2025.07.03.663076 (PMC12236518; doi:10.1101/2025.07.03.663076)
Supplement: Supplementary file 1 [file media-1.pdf]

Supporting Information

A Pipeline for Screening Small Molecule Enhanced Protein Stability in A Bacterial Orphan Receptor

James J. Siclari, Denize C. Favaro, Richard H. Huang, Kevin H. Gardner

Contents:

Figures S1-S5

Tables S1, S2

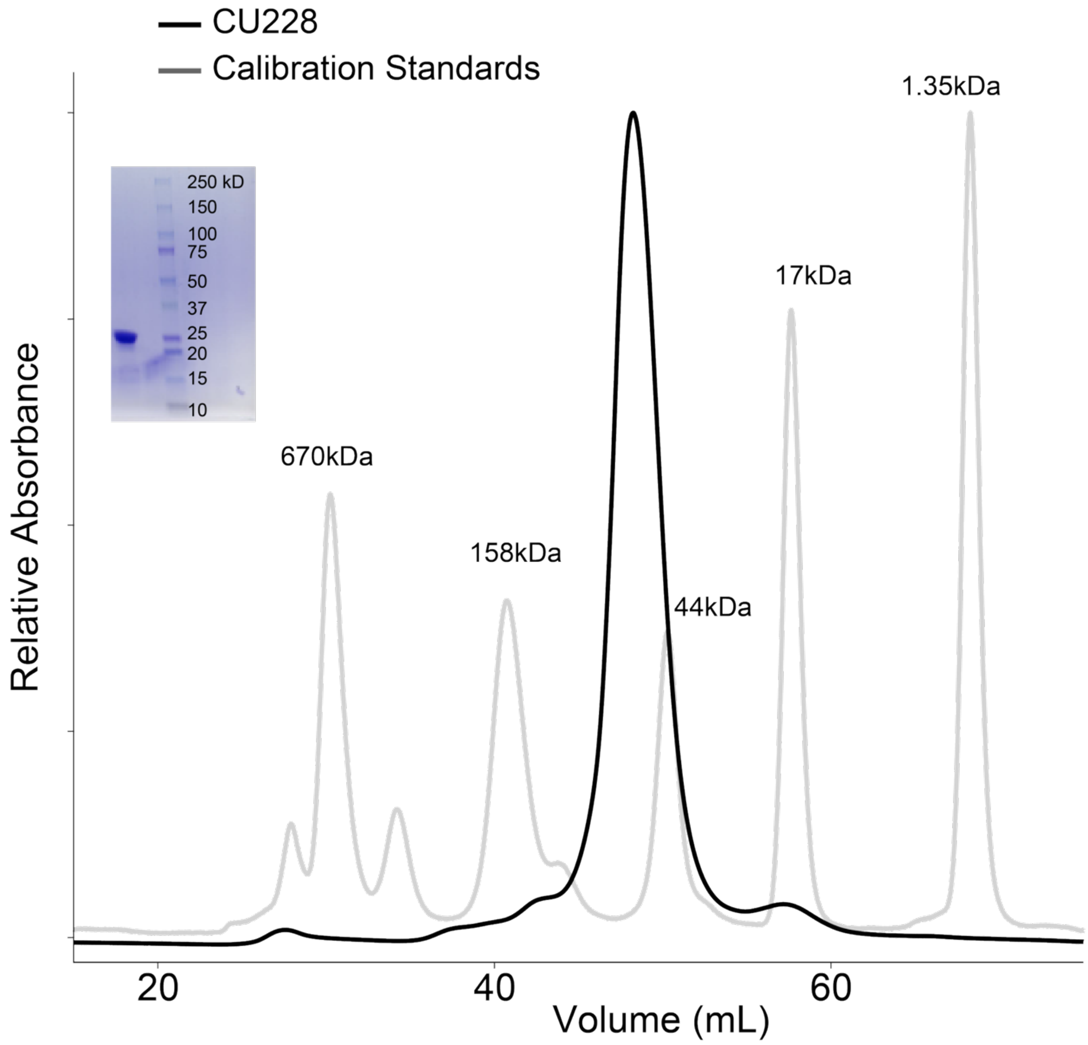

Figure S1. CU228 purification. SEC chromatogram of CU228 (black) and chromatography standards (grey). The insert shows SDS-PAGE gel of purified CU228.

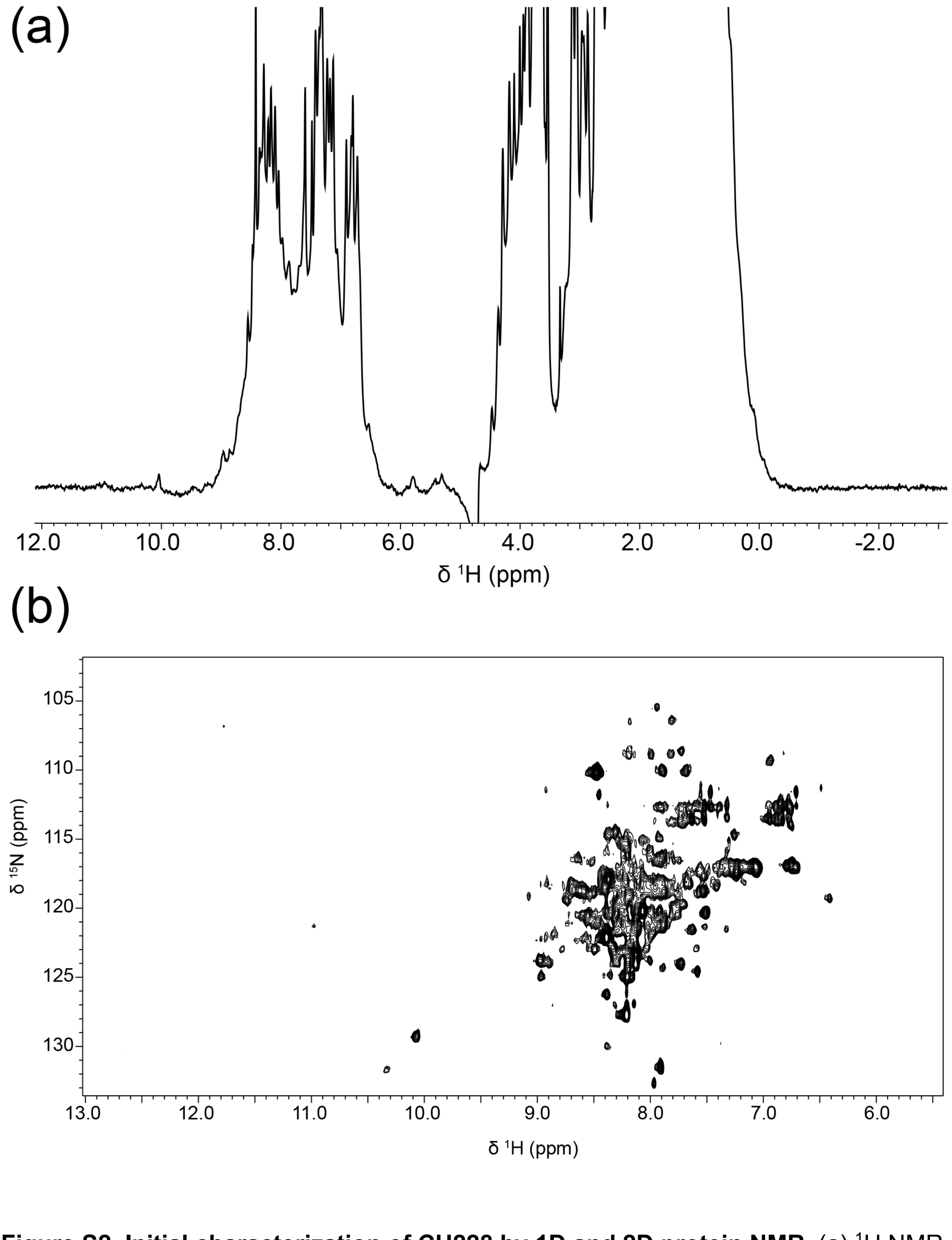

Figure S2. Initial characterization of CU228 by 1D and 2D protein NMR. (a) ^{1}H NMR of CU228. (b) $^{1}H/^{15}N$ HSQC TROSY of CU228.

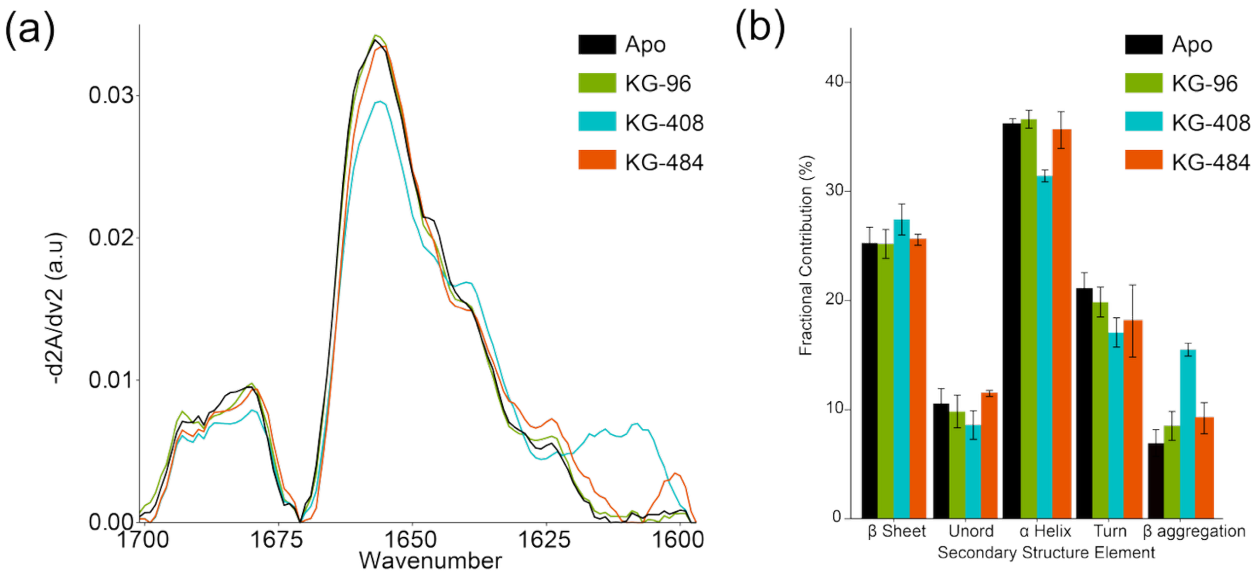

Figure S3. MMS of CU228 with small molecules at 25°C. (a) Second derivative Gaussian fits of IR spectra recorded for Apo CU228 and CU228 with 250 μM KG-96, KG-408, and KG-484. (b) Higher-order structure fractional contribution of each sample calculated from traces in panel a.

+KG-96

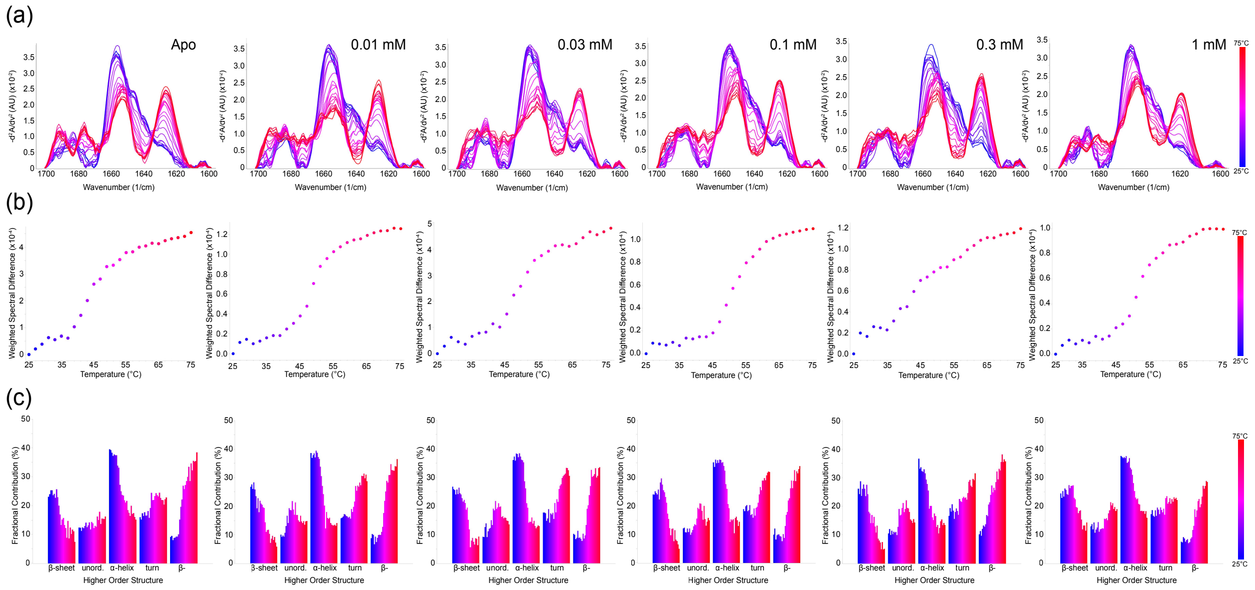

Figure S4. Concentration Dependent Thermal ramp MMS of CU228 and KG-96. (a) IR Traces across from 25-75°C at five different concentrations. (b) Weighted spectral difference across the thermal ramp range at each concentration. (c) Higher order structure fractional contribution as a function of temperature for each titration point.

+KG-484

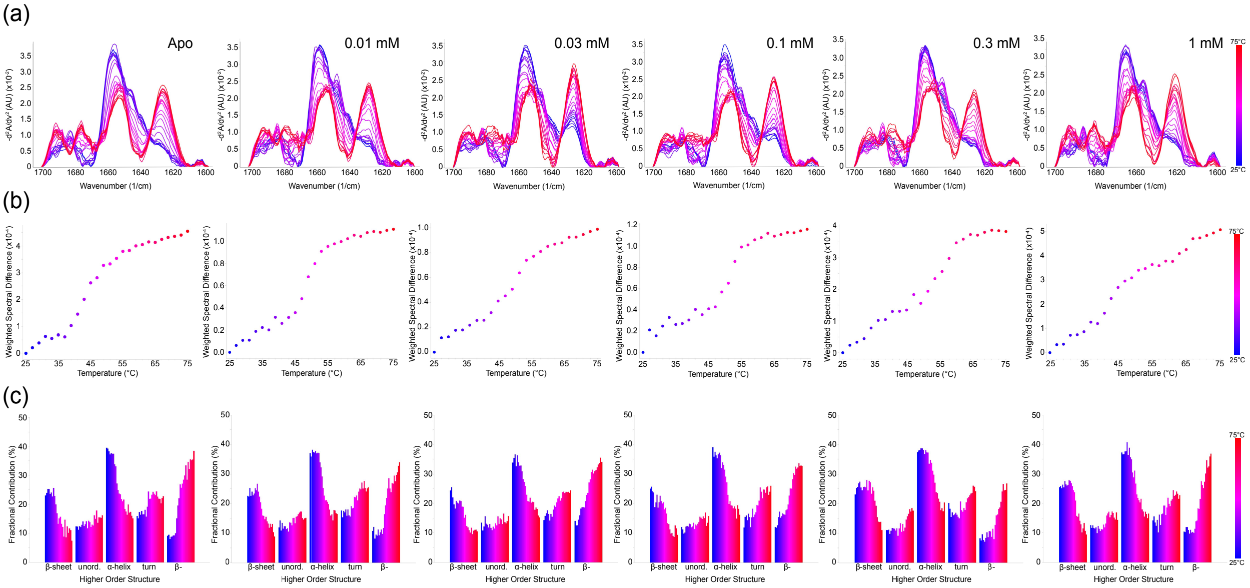

Figure S5. Concentration Dependent Thermal ramp MMS of CU228 and KG-484. (a) IR Traces across from 25-75°C at five different concentrations. (b) Weighted spectral difference across the thermal ramp range at each concentration. (c) Higher order structure fractional contribution as a function of temperature for each titration point.

Wavenumber (cm^{-1})	Secondary Structure
1618	Beta- (intermolecular β-sheet)
1624	Beta-
1627	Beta-
1632	Beta
1638	Beta
1642	Beta
1650	Unordered
1656	Alpha
1666	Turn
1672	Turn
1680	Turn
1688	Turn
1695	Beta-

Table S1. Peak assignments for Gaussian curve fitting.

Ligand	[Ligand] (μM)	T_m (β-)	T_m (β-sheet)	T_m (α-helix)
**Apo**	-	48.3	47.0	43.3
**KG-96**	0.01	49.0	49.6	47.2
	0.03	51.3	52.3	47.2
	0.1	51.8	54.6	47.3
	0.3	42.3	53.9	44.1
	1	53.4	60.0	47.8
**KG-408**	0.01	48.6	49.2	44.3
	0.03	51.9	51.8	45.7
	0.1	51.7	53.3	45.1
	0.3	53.7	55.4	45.0
	1	54.4	55.3	46.5
**KG-484**	0.01	50.5	51.2	46.7
	0.03	49.4	52.0	47.8
	0.1	53.4	55.7	51.1
	0.3	55.7	60.0	52.5
	1	53.3	61.0	47.5

Table S2: Secondary structure melting temperatures (T_m) of CU228 with and without small molecule ligands. KG-96, KG-408, and KG-484 were added at five different concentrations as indicated. (β- = intermolecular β-sheet aggregation)

Ligand	Peak Region	[Ligand] (μM)	STD-AF_{max}	k_{sat} (s^{-1})	STD-AF_0 (STD-AF_{max} x k_{sat})
KG96	1	0	-	-	0
		200	1.16	0.0632	0.073312
		400	0.744	0.123	0.091512
		600	1.39	0.066	0.09174
		800	1.22	0.0763	0.093086
		1000	0.52	0.232	0.12064
	2	0	-	-	0
		200	1.75	0.0578	0.10115
		400	1.26	0.11	0.1386
		600	1.9	0.0882	0.16758
		800	1.26	0.142	0.17892
		1000	1.28	0.156	0.19968
	3	0	-	-	0
		200	0.912	0.212	0.193344
		400	1.08	0.187	0.20196
		600	1.65	0.129	0.21285
		800	1.16	0.207	0.24012
		1000	1.18	0.21	0.2478
KG408	1	0	-	-	0
		200	1.21	0.513	0.62073
		400	2.33	0.426	0.99258
		600	3.52	0.336	1.18272
		800	4.09	0.296	1.21064
		1000	4.73	0.327	1.54671
	2	0	-	-	0
		200	1.57	0.505	0.79285
		400	3.03	0.41	1.2423
		600	3.83	0.367	1.40561
		800	4.41	0.362	1.59642
		1000	5.44	0.302	1.64288
	3	0	-	-	0
		200	1.36	0.508	0.69088
		400	2.61	0.43	1.1223
		600	3.57	0.389	1.38873
		800	4.39	0.328	1.43992
		1000	5.27	0.313	1.64951
KG484	1	0	-	-	0
		200	0.0747	0.821	0.0613287
		400	0.326	0.113	0.036838
		600	0.123	0.844	0.103812
		800	0.259	0.308	0.079772

		1000	0.185	0.62	0.1147
	2	0	-	-	0
		200	9.48	0.00214	0.0202872
		400	0.246	0.16	0.03936
		600	1.21	0.0274	0.033154
		800	1.76	0.0254	0.044704
		1000	0.533	0.111	0.059163
	3	0	-	-	0
		200	0.0552	0.276	0.0152352
		400	11.9	0.00178	0.021182
		600	0.178	0.155	0.02759
		800	6.91	0.00418	0.0288838
		1000	0.276	0.152	0.041952

Table S3: Variables derived from STD buildup curves (**Eq. 2**) used to plots Langmuir fittings.
